# Screening and Fermentation Parameter Optimization of Lipase-Producing *Trichosporon asahii*

**DOI:** 10.3390/microorganisms14081690

**Published:** 2026-08-01

**Authors:** Feng Li, Shuai Li, Jiaxin Li, Yuan He, Bin Deng, Haifeng Xiao

**Affiliations:** Hunan Provincial Key Laboratory of Xiangnan Rare-Precious Metals Compounds Research and Application, School of Chemistry and Environmental Science, Xiangnan University, Chenzhou 423000, China; lifeng214@xnu.edu.cn (F.L.); lishuai1218@xnu.edu.cn (S.L.); nora123456nice@163.com (J.L.); yuanhe8956@163.com (Y.H.)

**Keywords:** lipase, *Trichosporon asahii*, enzymatic properties, fermentation optimization, response surface methodology

## Abstract

Waste cooking oil is generated in large quantities during food-waste processing, and its complex composition may pose environmental and public-health risks if it is improperly managed. Lipase-mediated conversion of waste cooking oil into biodiesel offers a promising strategy for both waste-oil valorization and greener fuel production. To expand the available microbial sources of lipases, this study isolated a strain with strong lipase-producing capacity from composted food-waste samples. The isolate was identified using molecular biological methods, the enzymatic properties of the extracellular lipase were characterized, and fermentation conditions for enzyme production were optimized by response surface methodology. The strain was identified as *Trichosporon asahii*. The lipase showed optimal catalytic activity at 40 °C and pH 8.0. Na^+^ and K^+^ enhanced lipase activity, whereas Ca^2+^, Mg^2+^, Mn^2+^, and Fe^3+^ inhibited the enzyme. Response surface optimization showed that a maximum lipase activity of 70.816 U/mL was obtained at a fermentation temperature of 31 °C, an initial pH of 6.33, an inoculum size of 6.843%, and a fermentation time of 120 h, representing an approximately 10-fold increase compared with the non-optimized condition. These findings provide a useful basis for developing food-waste-derived oils as biodiesel feedstocks through lipase-based fermentation and support the resource-oriented utilization of food waste.

## 1. Introduction

Lipases (EC 3.1.1.3) hydrolyze triglycerides and catalyze esterification, transesterification, alcoholysis, and related reactions, supporting their use in food processing, environmental remediation, and biodiesel synthesis [1,2,3]. Microbial lipases stand out over animal and plant-derived counterparts owing to broad substrate tolerance, high yield and low cultivation costs, without seasonal production limitations [4].

Waste cooking oil, accounting for ~10% of kitchen waste mass, consists of long-chain triglycerides, diglycerides and free fatty acids formed during high-temperature frying [5]. Improper disposal may create environmental and public-health risks, whereas its lipid-rich composition makes it a low-cost, non-edible feedstock for biodiesel production [6,7,8]. Biodiesel is mainly produced through transesterification, which is commonly catalyzed by acids, bases, or enzymes [9]. Compared with acid- or base-catalyzed processes, lipase-catalyzed conversion can proceed under milder conditions, generate fewer by-products, and better accommodate feedstocks with high free-fatty-acid contents, although enzyme cost and insufficient operational performance remain important constraints [10,11,12,13,14]. Microbial lipase production is strongly strain-dependent and is influenced by medium composition and cultivation variables such as pH, temperature, inoculum size, and fermentation time [15,16,17,18]. Consequently, screening microorganisms from lipid-rich environments is essential for identifying strains that combine high extracellular lipase productivity with tolerance to complex waste-oil matrices. Although lipase production by *Trichosporon asahii* has previously been demonstrated and enhanced through statistical medium optimization [19], food-waste-derived isolates have rarely been examined through an integrated workflow that combines quantitative strain screening, enzymatic properties, and systematic optimization of physical fermentation parameters. The specific novelty of this study therefore lies in isolating a lipid-adapted *Trichosporon asahii* strain from composted kitchen waste and linking its extracellular lipase properties with a four-factor fermentation optimization strategy, rather than merely demonstrating microbial lipase production.

We hypothesized that the lipid-rich microenvironment of composted food waste selects for microorganisms with high extracellular lipase-producing capacity, and that coordinated optimization of fermentation temperature, initial pH, inoculum size, and cultivation time would significantly enhance enzyme activity. Accordingly, the objective of this study was to isolate and quantitatively screen lipase-producing microorganisms from composted food waste; identify the selected strain by morphological and molecular analyses; characterize the temperature, pH, stability, and metal-ion responses of its extracellular lipase; and optimize and experimentally validate the principal physical fermentation parameters using single-factor experiments and a Box-Behnken response surface design. These results were expected to expand the available microbial resources for lipase production and provide a foundation for the industrial production of biodiesel from waste cooking oil through microbial enzymatic processes.

## 2. Materials and Methods

### 2.1. Primary Screening, Isolation, and Purification of Lipase-Producing Strains

Food waste (500 g) was collected from a swill container at the Fourth Canteen of Xiangnan University and incubated in a constant-temperature incubator at 37 °C for one week to allow decomposition. A 10 mL sample was collected from the oil-water interface of the rancid material and fully dispersed in 90 mL of sterile normal saline for 30 min. Subsequently, 5 mL of the upper mixed suspension was added to 25 mL of enrichment medium containing 0.1% ammonium nitrate, 0.01% dipotassium hydrogen phosphate, 0.15% ammonium sulfate, 0.05% magnesium sulfate heptahydrate, and 1.0% olive oil. The initial pH of the enrichment medium was adjusted to 7.0. The culture was then incubated at 37 °C and 200 rpm for 48 h.

A 0.1 mL aliquot of the enriched culture was transferred into a sterile tube containing 0.9 mL of sterile water, mixed thoroughly, and serially diluted 10-fold to obtain suspensions from 10^−1^ to 10^−6^. The 10^−4^, 10^−5^, and 10^−6^ dilutions were spread on tributyrin agar plates and incubated at 37 °C until colonies producing clear hydrolysis zones appeared. Colonies with large and distinct transparent zones were selected, numbered, and purified by streaking on tributyrin agar plates to obtain single colonies.

### 2.2. Lipase Activity Screening and Enzymatic Activity Determination

The backs of tributyrin agar plates were divided into four equal areas by marking a cross with a marker pen. Sterile toothpicks were then used to pick monoclonal colonies of different isolates obtained by streak isolation and inoculate them onto the center of the corresponding areas. The inoculated plates were incubated at 37 °C for 24–36 h until clear zones appeared around all colonies. Lipase-producing capacity was preliminarily evaluated by calculating the ratio of the hydrolysis-zone diameter (D) to the colony diameter (d).

Colonies preserved on slants were reactivated in liquid medium, inoculated into fermentation medium, and incubated in a constant-temperature shaker at 37 °C and 200 rpm for 30 h. The fermentation broth was centrifuged at 5500 rpm and 4 °C for 20 min to obtain crude enzyme solution. Lipase activity was determined using the p-nitrophenyl palmitate (pNPP) method. In this assay, lipase hydrolyzes the ester bond of pNPP, a synthetic chromogenic substrate, producing palmitic acid and p-nitrophenol (pNP). Under alkaline conditions, pNP appears yellow and shows strong absorbance at 405 nm [3]. The rate of pNP formation is directly proportional to the amount of active lipase present in the sample.

Preparation of the pNP standard curve: A 1.0 mL aliquot of the stock solution was diluted to prepare a 10 μg/mL standard working solution. Then, 1 mL portions of the working solution were transferred into five 10 mL EP tubes and further diluted to final concentrations of 1, 2, 4, 6, and 8 μg/mL. The absorbance of each solution was measured at 405 nm. The standard curve was constructed using absorbance as the x-axis and concentration as the y-axis.

The determination procedure for lipase activity of samples was as follows: 30 mg of pNPP was dissolved in 10 mL of methanol. For the reaction mixture, 2.1 mL of Tris-HCl buffer (pH 8.0), 200 μL of the pNPP solution, and 100 μL of extracellular enzyme solution were added sequentially to 5 mL EP tubes. The mixture was incubated in a water bath at 40 °C for 10 min. The reaction was then terminated by adding 100 μL of 0.1 mol/L ZnSO_4_ solution, followed by cooling in an ice bath for 5–10 min. After filtration through a 0.22 μm aqueous filter membrane, the absorbance of the filtrate was measured at 405 nm.

One unit of enzyme activity was defined as the amount of enzyme required to release 1 μg of p-nitrophenol from the substrate per minute. The calculation formula for lipase activity is shown in Equation (1).

Lipase activity (U/mL) = [(A_2_ − A_1_) × V_1_]/(K × V_2_ × T) (1)

where A_1_ is the absorbance of the inactivated enzyme sample, A_2_ is the absorbance of the non-inactivated enzyme sample, K is the slope of the standard curve, V_1_ is the final volume of the reaction mixture, V_2_ is the volume of enzyme solution, and T is the reaction time.

### 2.3. Morphological Observation of the Strain

The obtained strains were diluted and spread onto YPD solid medium, followed by incubation at 37 °C for 24 h. The morphological characteristics of colonies, including shape, size, margin, color and luster, were observed, and the microscopic morphology of the strains was examined using a scanning electron microscope (SEM).

### 2.4. Molecular Biological Identification

After the target strain was cultured to the logarithmic phase, samples were submitted to Sangon Biotech (Shanghai) Co., Ltd. (Shanghai, China) for sequencing of the 26S rRNA D1/D2 region. The resulting sequence was compared with reference sequences in the NCBI database using BLAST (version 2.14.1+). A phylogenetic tree was then constructed in combination with the morphological characteristics of the strain.

### 2.5. Enzymatic Characterization of Lipase

After activation, the strain was transferred into fermentation medium to obtain crude enzyme solution. Lipase activity was measured under different conditions to determine the optimal reaction conditions of the enzyme.

(1) At pH 8.0, lipase activity was measured at 30, 35, 40, 45, and 50 °C, and thermal stability was evaluated.
(2) At 40 °C, lipase activity was measured at pH 5.0, 6.0, 7.0, 8.0, and 9.0, and pH stability was evaluated.
(3) Under the optimal pH and temperature conditions, different metal ions (Na^+^, K^+^, Mg^2+^, Ca^2+^, Mn^2+^, and Fe^3+^) were added to the reaction system to a final concentration of 10 mM, and lipase activity was measured.

### 2.6. Optimization of Fermentation Conditions

#### 2.6.1. Single-Factor Optimization

(1) Effect of fermentation pH. *Trichosporon asahii* was inoculated into 25 mL of fermentation medium at an inoculum size of 2%. The initial pH of the medium was adjusted to 5.0, 6.0, 7.0, 8.0, or 9.0. Cultures were incubated at 30 °C and 200 rpm for 72 h. The crude enzyme solution was collected by centrifugation, and lipase activity was measured to determine the optimal fermentation pH.
(2) Effect of fermentation time. *Trichosporon asahii* was inoculated into 25 mL of fermentation medium at an inoculum size of 2%. At the optimal pH, cultures were incubated at 30 °C and 200 rpm for 24, 48, 72, 96, or 120 h. Lipase activity in the crude enzyme solution was measured to determine the optimal culture time.
(3) Effect of inoculum size. Under the optimal initial pH, *Trichosporon asahii* was inoculated into 100 mL Erlenmeyer flasks containing 25 mL of fermentation medium at inoculum sizes of 2%, 4%, 6%, 8%, or 10%. Cultures were incubated at 30 °C and 200 rpm for the optimal fermentation time, and the crude enzyme solution was collected for lipase activity determination.
(4) Effect of culture temperature. Under the optimal fermentation pH and fermentation time, *Trichosporon asahii* was inoculated into 100 mL Erlenmeyer flasks containing 25 mL of fermentation medium at the optimal inoculum size. Cultures were incubated at 25 °C, 28 °C, 30 °C, 32 °C, or 35 °C at 200 rpm for the optimal fermentation time, and lipase activity was measured to determine the optimal culture temperature.

#### 2.6.2. Response Surface Methodology

Based on the single-factor experiments, a four-factor, three-level Box-Behnken design was used to systematically evaluate the effects of fermentation temperature (28, 30 and 32 °C), initial pH (5.0, 6.0 and 7.0), fermentation time (72, 96 and 120 h), and inoculum size (4.0, 6.0 and 8.0%), as well as their interactions, on lipase production by *Trichosporon asahii*. Lipase activity was used as the response variable.

### 2.7. Statistical Analysis

All lipase activity detection assays and fermentation experiments were conducted in triplicate, and experimental data were expressed as mean ± standard deviation (SD). Statistical analysis of single-factor experiments was carried out via one-way ANOVA and LSD multiple comparison tests using SPSS Statistics 25 software (IBM Corporation, Armonk, NY, USA). Response surface regression modeling for fermentation optimization was completed with Design-Expert 13 software (Stat-Ease Inc., Minneapolis, MN, USA).

## 3. Results

### 3.1. Primary Screening of Lipase-Producing Strains

**Figure 1:**
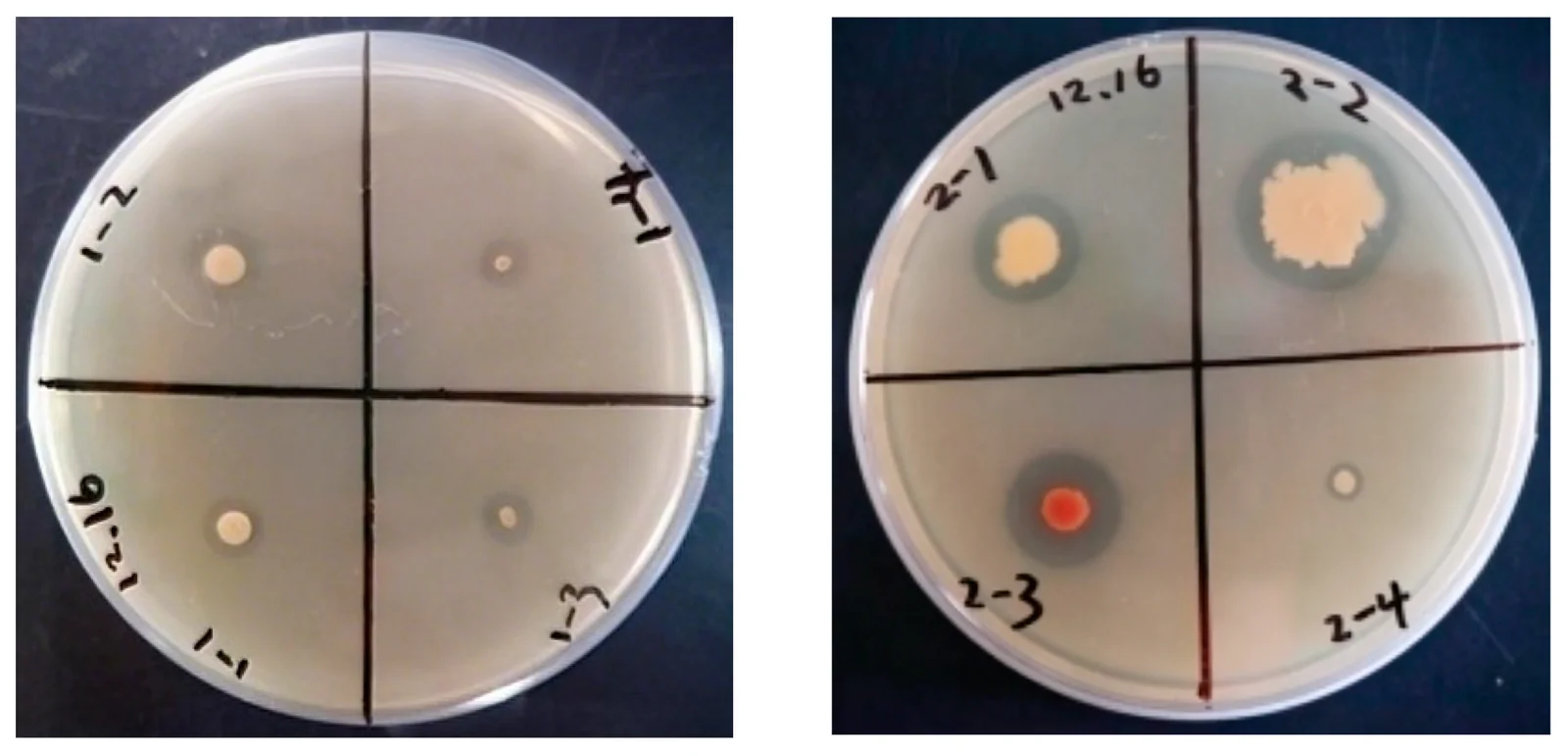
Hydrolysis zones of lipase-producing strains.

After primary screening, eight lipase-producing isolates that formed obvious hydrolysis zones were obtained. The hydrolysis zones produced by the isolates are shown in Figure 1. Eight lipase-positive isolates were obtained and designated as strains 1-1, 1-2, 1-3, 1-4, 2-1, 2-2, 2-3, and 2-4. Because strains 1-1 and 1-2, as well as strains 1-3 and 1-4, were indistinguishable in terms of colony and cellular morphology, they were considered duplicate isolates. Consequently, six representative strains—1-2, 1-4, 2-1, 2-2, 2-3, and 2-4—were selected for secondary screening and quantitative determination of lipase activity.

### 3.2. Secondary Screening of Lipase-Producing Strains

The standard curve equation for p-nitrophenol was y = 0.13634x + 0.03037 (y: p-nitrophenol concentration (μg/mL); x: OD_405_ after color development). With a slope of 0.13634 and an R^2^ value of 0.9995, an excellent linear relationship was observed between concentration and absorbance. This equation was applied for the calculation of lipase activity.

The D/d ratios and enzyme activities are shown in Table 1; strains 2-3 formed a large tributyrin hydrolysis halo with a D/d value of 2.2 on agar plates, yet strains 2-1 to 2-4 all showed drastically lower lipase activity in olive oil liquid culture compared to strains 1-2 and 1-4. Tributyrin plate screening only reflects short-chain esterase activity rather than the strain’s true ability to decompose long-chain triglycerides in olive oil, which explains the mismatch between large halos and poor liquid fermentation performance of strains 2-3. Given their low lipase activities and unstable fermentation traits, all four strains were discarded.

Strains 1-4 showed the highest overall lipase activity (9.290 U/mL); however, they were identified as *Ralstonia pickettii*, a high-risk opportunistic pathogen that poses severe biosafety hazards for industrial fermentation. Therefore, strains 1-2 with the second-highest lipase activity (7.165 U/mL) were selected as the target strains for follow-up experiments. Although *Trichosporon asahii* is occasionally reported as an opportunistic pathogen, its infection incidents only occur in severely immunocompromised groups, and standardized closed fermentation operations can fully control biosafety risks. Moreover, strains 1-2 maintained stable growth and consistent lipase production with olive oil as a carbon source in repeated cultures, satisfying the requirements for systematic enzymatic characterization and fermentation optimization.

### 3.3. Identification and Phylogenetic Analysis of Strains 1-2

**Figure 2:**
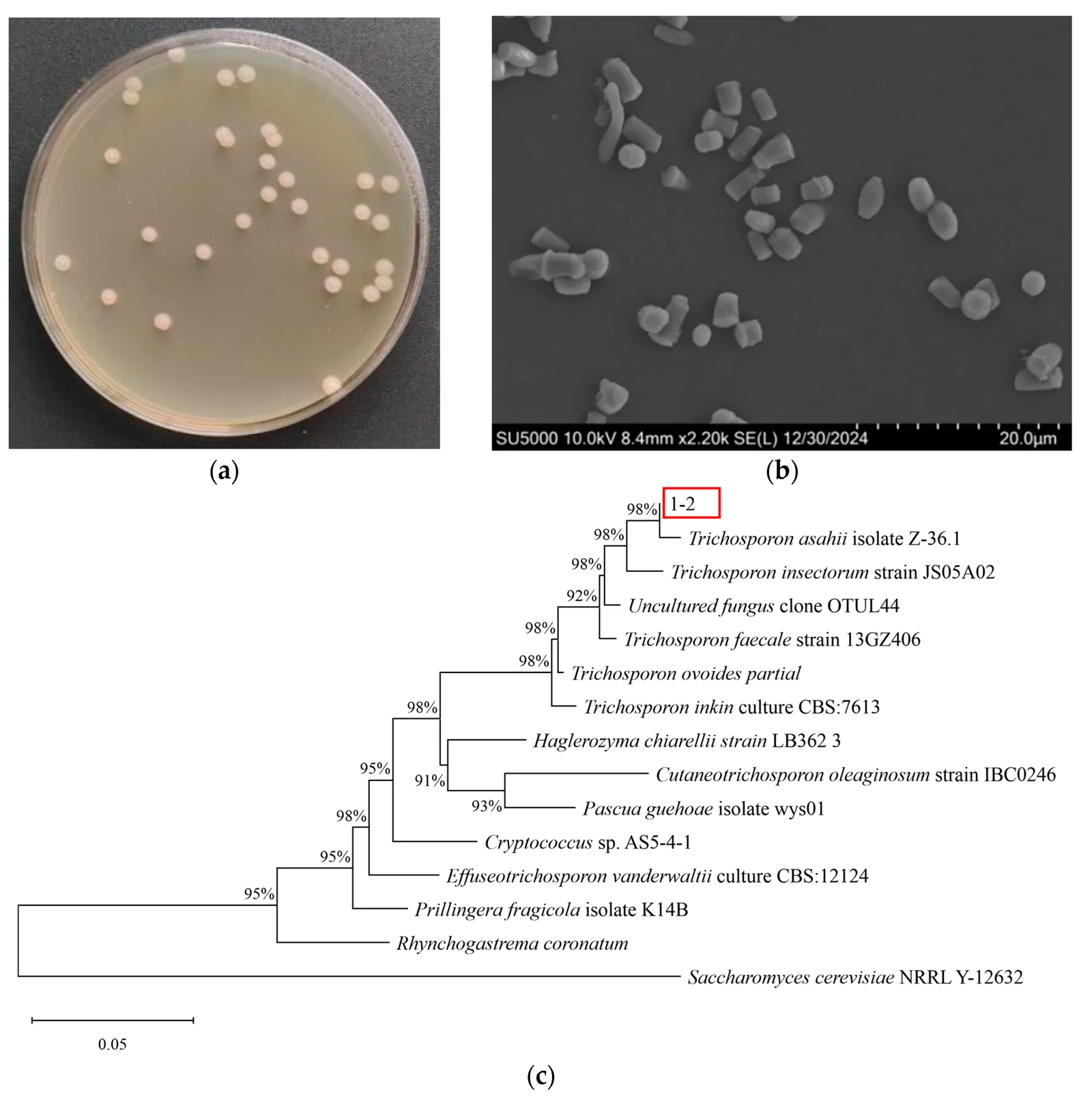
Morphological observation of strains 1-2: (**a**) colony morphology; (**b**) SEM image (2000×); (**c**) phylogenetic tree of strains 1-2. Numbers 1-2 within the red square denote strains 1-2.

After 24–48 h of cultivation on YPD solid medium, strains 1-2 formed milky-white, punctate colonies that were smooth, moist, and raised. After 72 h of incubation, the colonies became larger (Figure 2a), with a dry texture, central protuberance, and folded surface. The colonies had irregular serrated edges, and short white flocculent hyphae were observed around them. SEM images are shown in Figure 2b. At 2000× magnification, the cells had a major-axis length of approximately 40 μm. Individual cells were short rod-shaped or oval, and a small number of budding spores were observed.

The 26S rRNA D1/D2 region of the target strain was sequenced, yielding a 646 bp sequence. BLAST analysis showed that the isolate shared nearly 100% sequence similarity with *Trichosporon asahii*. The phylogenetic tree (Figure 2c) further confirmed that strains 1-2 belonged to *Trichosporon asahii*.

### 3.4. Enzymatic Properties of Lipase Produced by Trichosporon asahii

#### 3.4.1. Optimal Reaction Temperature and Thermal Stability

**Figure 3:**
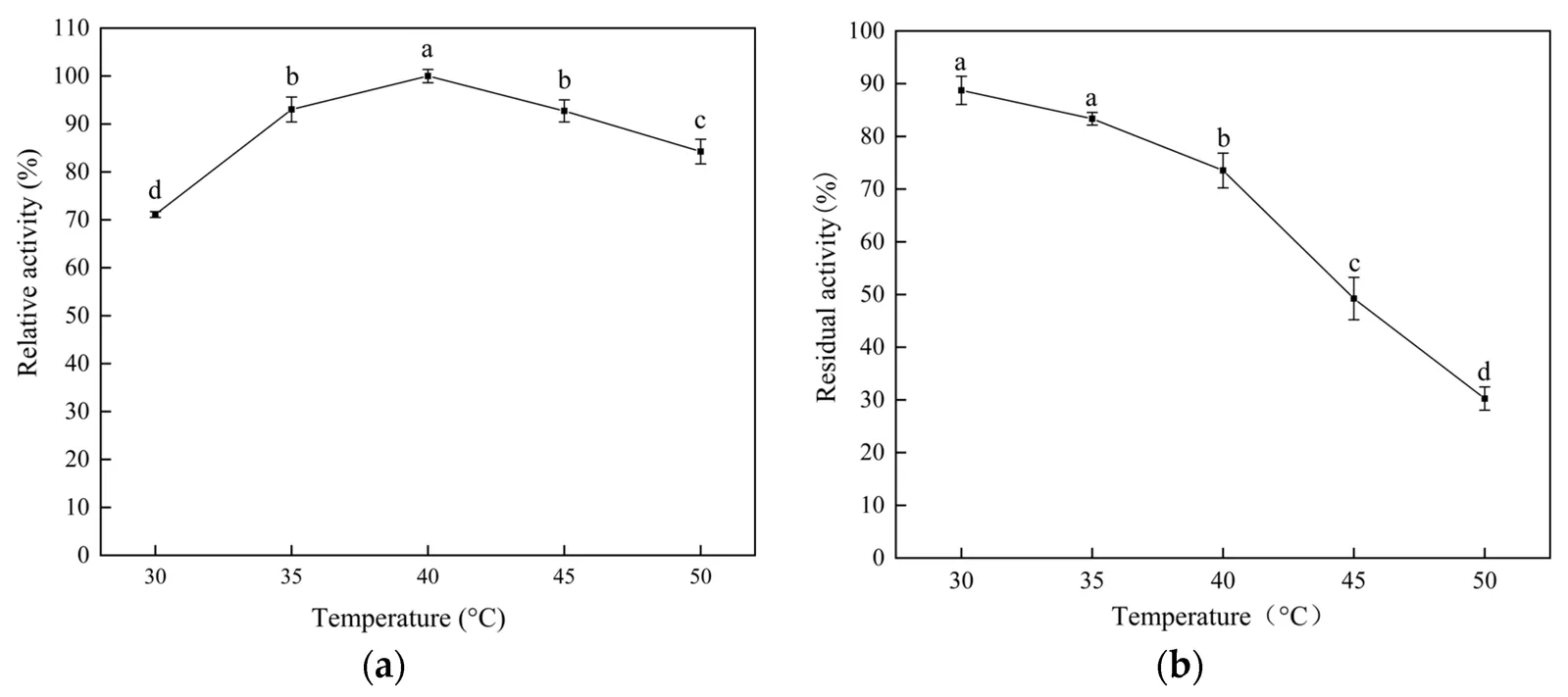
Optimal reaction temperature and thermal stability of lipase: (**a**) optimal reaction temperature; (**b**) thermal stability. Relative activity is calculated with the maximum enzyme activity defined as 100%. Residual activity represents the activity retained after 2 h incubation at corresponding temperatures, with the activity of untreated crude lipase set as 100%. Different lowercase letters indicate significant differences (*p* < 0.05).

The optimal reaction temperature of the lipase is shown in Figure 3a. Within 30–40 °C, the relative enzyme activity increased with increasing temperature and reached its maximum at 40 °C. When the temperature exceeded 40 °C, lipase activity decreased. These results indicated that the lipase produced by this strain was a mesophilic enzyme with an optimal reaction temperature of 40 °C. Similar optimal temperatures (35–40 °C) have been reported for many microbial lipases derived from yeasts and filamentous fungi [18].

The thermal stability of the lipase is shown in Figure 3b. The crude enzyme retained over 80% residual activity after incubation at 30–35 °C for 2 h, indicating favorable stability under mild temperature conditions. As temperature rose continuously, the residual activity declined progressively, which was attributed to thermal unfolding and irreversible denaturation of lipase protein structure at high temperatures. Temperature is a critical factor influencing enzyme structure and function, as elevated temperatures enhance molecular motion and may disrupt non-covalent interactions stabilizing the protein’s tertiary structure, leading to partial unfolding or irreversible denaturation and consequent loss of catalytic activity [20,21]. In contrast, lower temperatures help maintain structural integrity and preserve enzyme activity over time.

#### 3.4.2. Optimal Reaction pH and pH Stability

**Figure 4:**
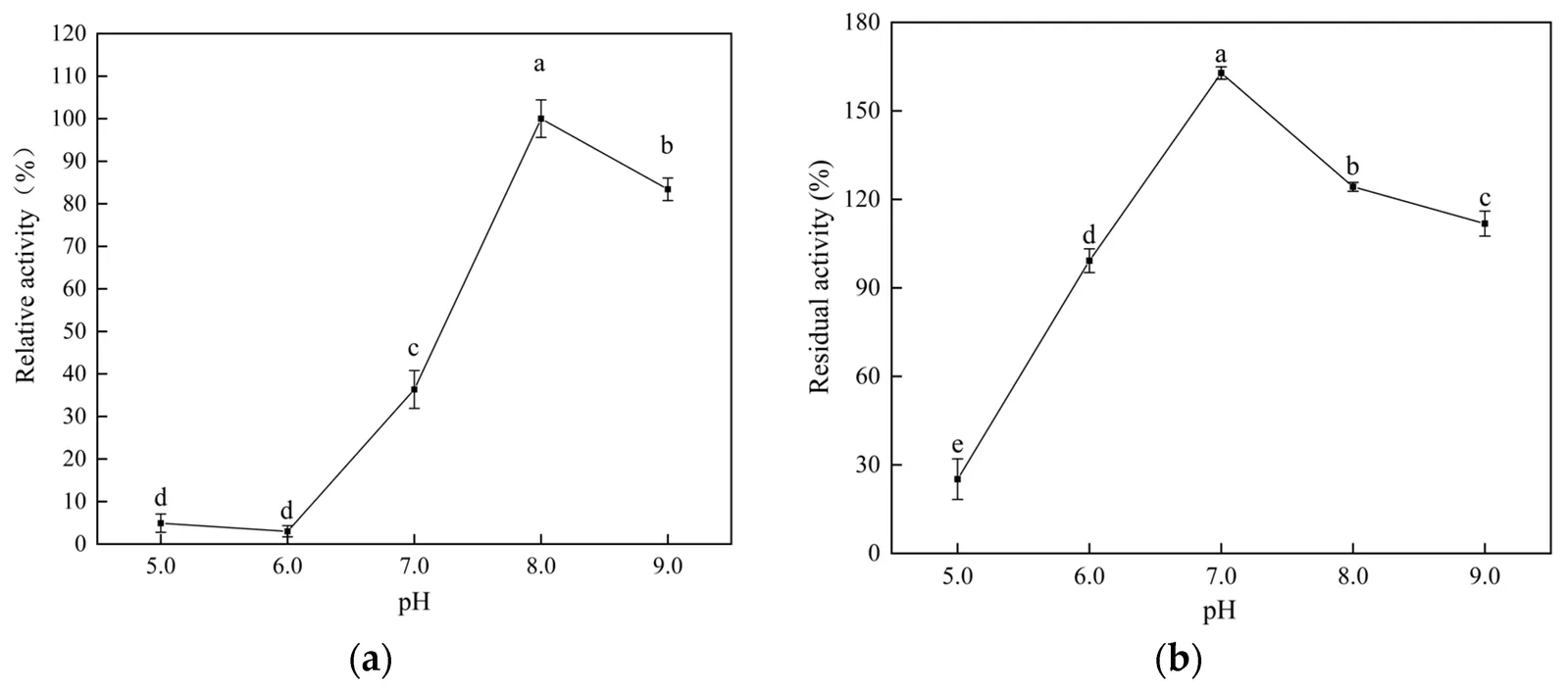
Optimal reaction pH and pH stability of lipase: (**a**) optimal reaction pH; (**b**) pH stability. Relative activity is calculated with the maximum enzyme activity defined as 100%. Residual activity represents the activity retained after pre-incubation at corresponding pH values, with the activity of untreated crude lipase set as 100%. Different lowercase letters indicate significant differences (*p* < 0.05).

The optimal reaction pH of the lipase is shown in Figure 4a. The enzyme exhibited low activity at pH 5.0–6.0. As the pH increased from 6.0 to 8.0, the relative enzyme activity increased markedly and reached a maximum at pH 8.0, indicating that the enzyme is an alkaline lipase. When the pH exceeded 8.0, enzyme activity gradually declined, suggesting reduced catalytic efficiency under strongly alkaline conditions. These results indicate that the enzyme is highly sensitive to reaction pH, which plays a critical role in modulating enzyme conformation and the electrostatic environment of the active site, thereby influencing substrate binding affinity and catalytic efficiency [20].

The pH stability profile (Figure 4b) revealed that the lipase retained only low residual activity under strongly acidic pH 5.0, while moderate residual activity was maintained at pH 6.0. Pre-incubation at pH 7.0–9.0 obviously elevated residual activity, with the highest residual activity observed at pH 7.0; this activation effect weakened as alkalinity increased. This non-classical stability behavior may be attributed to pH-induced changes in enzyme aggregation state or structural rearrangement, which could promote the formation of more catalytically active conformations in specific buffer environments [22].

#### 3.4.3. Effects of Metal Ions on Lipase Activity

**Figure 5:**
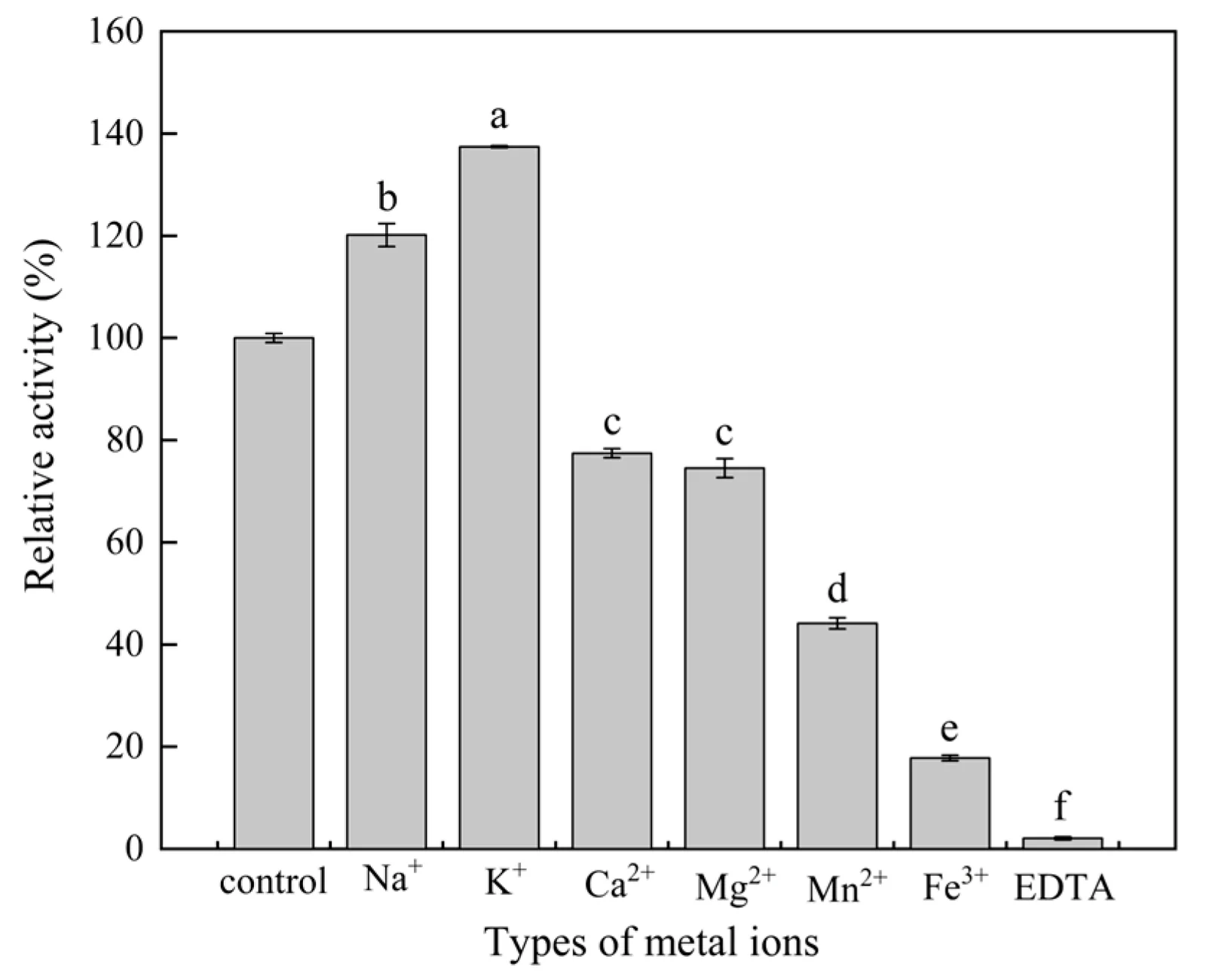
Effects of metal ions on lipase activity. Relative activity of lipase with the blank control group defined as 100%. Different lowercase letters indicate significant differences (*p* < 0.05).

The effects of metal ions on lipase activity are shown in Figure 5. As shown in the figure, different metal ions exerted distinct effects on lipase activity. Compared with the control, K^+^ significantly enhanced lipase activity and produced the strongest activation effect, increasing the relative activity to approximately 140%, followed by Na^+^, which increased enzyme activity to about 120%. In contrast, all tested divalent and trivalent metal ions inhibited lipase activity to varying degrees. Ca^2+^ and Mg^2+^ caused moderate inhibition, retaining approximately 70–80% of the original activity. Mn^2+^ exhibited a stronger inhibitory effect, reducing the relative activity to approximately 45%, whereas Fe^3+^ showed the strongest inhibition among the metal ions, leaving only about 20% residual activity. Notably, EDTA almost completely abolished lipase activity, indicating that metal ions are essential for maintaining the catalytic activity and structural integrity of the enzyme. Although monovalent cations usually do not act as enzyme cofactors, they may bind to negatively charged amino acid residues on the enzyme surface, facilitating opening of the lid domain [23] and promoting activation at the water-oil interface [24]. This increases the probability of collisions between the active center and the substrate, ultimately enhancing enzymatic activity. In contrast, the reduced activity after treatment with Ca^2+^, Mg^2+^, Mn^2+^, and Fe^3+^ may result from their binding to key amino acid residues in the enzyme, reducing the affinity of the active site for the substrate and thereby exerting an inhibitory effect [25].

### 3.5. Effects of Fermentation Conditions on Lipase Activity by Trichosporon asahii

**Figure 6:**
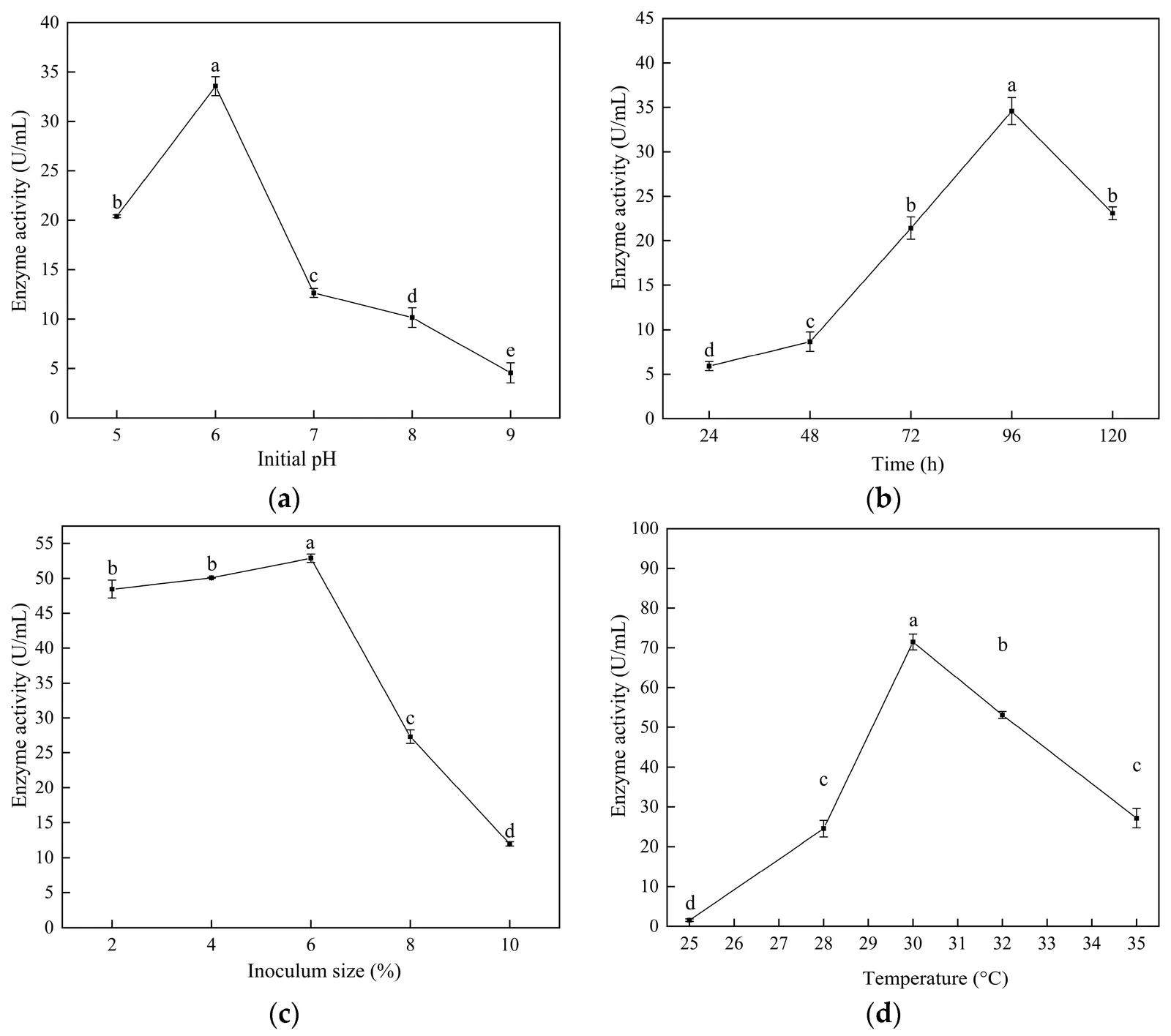
Effects of fermentation conditions on lipase production: (**a**) initial pH; (**b**) fermentation time; (**c**) inoculum size; (**d**) fermentation temperature. Different lowercase letters indicate significant differences (*p* < 0.05).

The effect of fermentation pH on lipase activity by *Trichosporon asahii* is shown in Figure 6a. Lipase activity increased as pH rose from 5.0 to 6.0 and reached a maximum of 33.571 U/mL at pH 6.0. When pH exceeded 6.0, enzyme activity decreased sharply and then gradually declined under alkaline conditions (pH 7.0–9.0). This suggests that *Trichosporon asahii* prefers slightly acidic conditions for optimal growth and enzyme synthesis. pH is a key environmental factor influencing microbial metabolism by regulating membrane transport processes, enzyme expression, and intracellular homeostasis. Deviation from the optimal pH range may suppress microbial growth or reduce enzyme biosynthesis efficiency.

The effect of fermentation time on lipase activity is shown in Figure 6b. Lipase activity increased steadily from 24 to 96 h, reaching a maximum of 36.296 U/mL at 96 h, followed by a decline at 120 h. This pattern indicates that lipase synthesis is growth-associated and primarily occurs during the late exponential to early stationary phase. The subsequent decline may be attributed to nutrient depletion and proteolytic degradation of extracellular enzymes during prolonged cultivation.

The effect of inoculum size on lipase activity is shown in Figure 6c. As shown in Figure 6c, increasing the inoculum size from 2% to 6% significantly enhanced lipase activity, which peaked at 52.884 U/mL at 6%. Further increase in inoculum size resulted in a marked decline in enzyme activity. This may be due to excessive biomass leading to rapid nutrient depletion and accumulation of inhibitory metabolites, which negatively affects both cell growth and enzyme secretion. Conversely, insufficient inoculum delays fermentation initiation and increases contamination risk.

The effect of temperature on lipase production is shown in Figure 6d. Lipase activity increased with temperature from 25 to 30 °C and reached a maximum of 71.440 U/mL at 30 °C. Further increases in temperature resulted in a significant decrease in enzyme production. Temperature strongly influences microbial growth rate, membrane fluidity, and enzyme biosynthesis. High temperature accelerates cellular aging and may induce metabolic stress, while low temperature reduces metabolic activity and enzyme synthesis efficiency.

### 3.6. Response Surface Optimization of Lipase Activity by Trichosporon asahii

#### 3.6.1. Establishment of the Regression Equation and Analysis of Variance

Response surface methodology was used to optimize four factors affecting lipase activity: fermentation temperature, initial pH, fermentation time, and inoculum size. A total of 29 experimental runs were performed, including 24 factorial-analysis runs and five center-point replicates. The data in Table 2 were analyzed using Design-Expert 13 software, and the regression equation is shown in Equation (2).

Y = 53 + 2.89A + 7.65B + 15.25C + 3.46D + 4.75AB + 9.57AC − 0.2197AD + 1.05BC − 1.81BD + 2.92CD − 14.38A^2^ − 15.72B^2^ − 2.06C^2^ − 6.76D^2^ (2)

The ANOVA results are presented in Table 3. The model was significant (*p* < 0.05), whereas the lack-of-fit term was not significant (*p >* 0.05), indicating that the model fitted the experimental data well and had high reliability. The coefficient of determination (R^2^) of the regression model was 0.7175, indicating a good fit between lipase activity and the fermentation conditions. The adequate precision of the model was 5.2678 (>4), further indicating that the model could be used to predict the response variable. According to the F-values of the four factors, the effects on lipase activity followed the order C > B > D > A; thus, fermentation time had the strongest effect, followed by pH, inoculum size, and fermentation temperature. The linear term C had a highly significant effect on lipase activity (*p* < 0.01), while the quadratic terms A^2^ and B^2^ were significant (*p* < 0.05).

#### 3.6.2. Interaction Effects Among Factors

**Figure 7:**
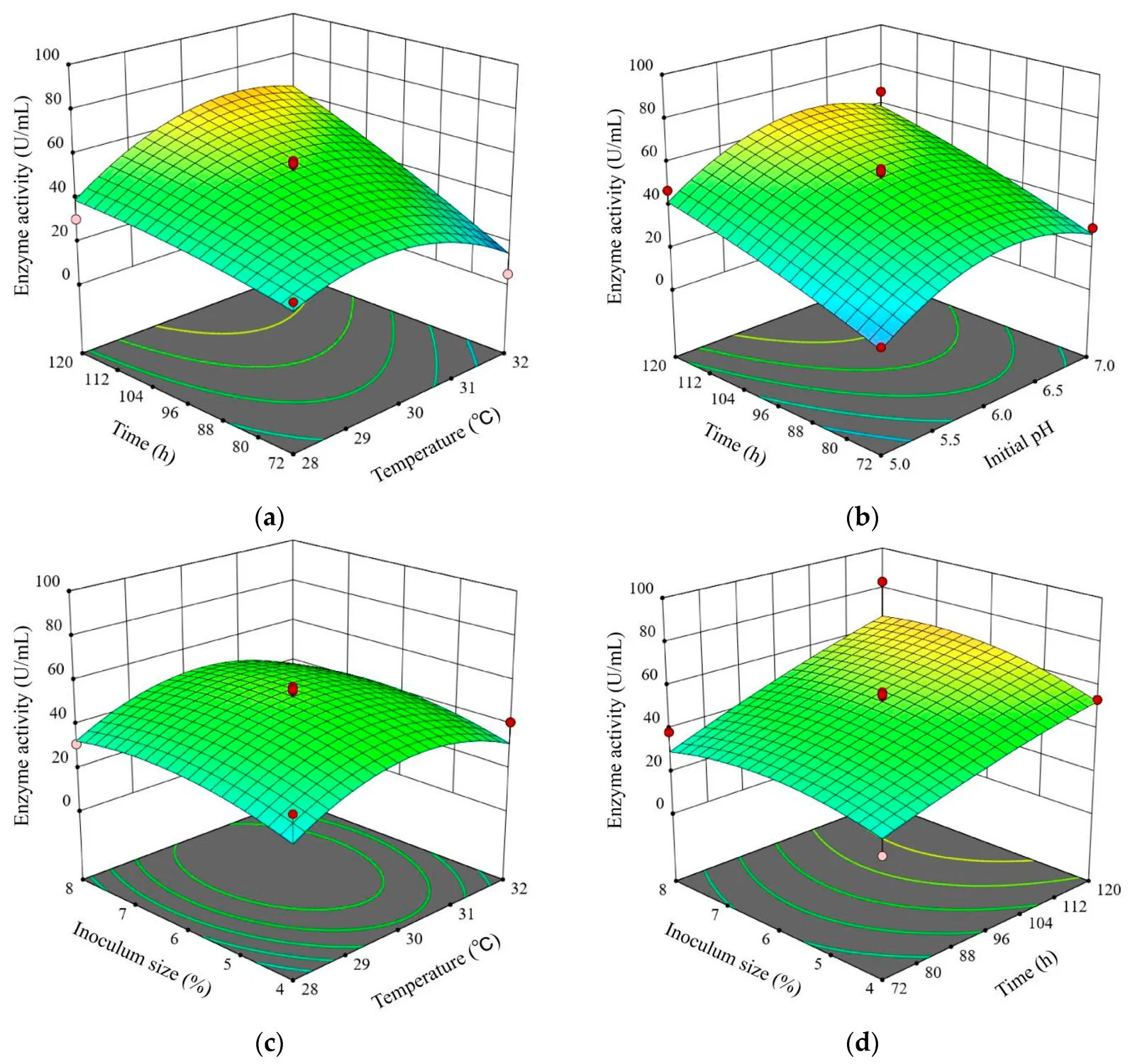

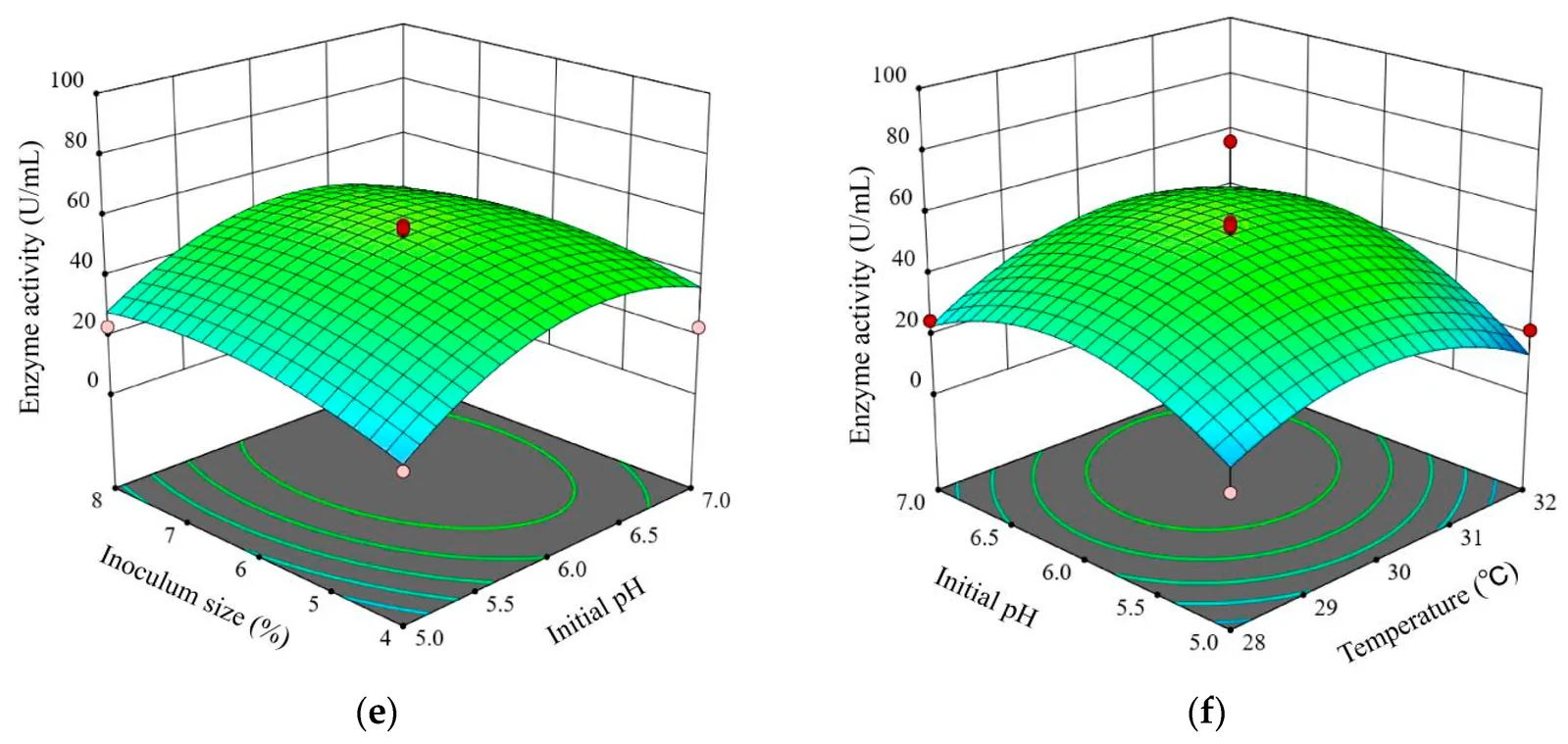
Response surface plots of lipase production under different fermentation conditions. Cool-to-warm color gradients are used to reflect the ascending trend of enzyme activity response values. Abundant color transitions between shades represent drastic fluctuations in response values. Specifically, dark blue stands for the minimum enzyme activity, green for the intermediate level, and bright yellow/orange yellow for the highest peak of enzyme activity. (**a**) Response surface plots for the interactive effects of fermentation time and temperature on lipase activity; (**b**) Response surface plots for the interactive effects of fermentation time and initial pH on lipase activity; (**c**) Response surface plots for the interactive effects of fermentation inoculum size and temperature on lipase activity; (**d**) Response surface plots for the interactive effects of fermentation inoculum size and fermentation time on lipase activity; (**e**) Response surface plots for the interactive effects of fermentation inoculum size and initial pH on lipase activity; (**f**) Response surface plots for the interactive effects of fermentation initial pH and temperature on lipase activity.

The response surface and contour plots showing the interaction effects of the four factors on lipase activity are presented in Figure 7. The color gradient in the response surfaces reflects the increasing trend of lipase activity. The convex shape of the surfaces indicates the presence of an optimum response value. In contour plots, a more elliptical distribution and greater curvature indicate stronger interaction effects between two factors. The response surface plots showed maxima for all pairwise factor interactions. The contours in Figure 7c,e,f were closer to ellipses, indicating stronger interaction effects between fermentation temperature and pH, fermentation temperature and inoculum size, and pH and inoculum size. These interactions had notable effects on lipase activity produced by *Trichosporon asahii*.

#### 3.6.3. Optimal Fermentation Conditions and Verification

The optimal combination of conditions predicted by the response surface model was a fermentation temperature of 30.964 °C, initial pH of 6.326, inoculum size of 6.843%, and fermentation time of 120 h, with a predicted lipase activity of 71.961 U/mL.

To verify the predictive ability and reliability of the regression model, the predicted conditions were adjusted for practical operation to 31 °C, pH 6.33, an inoculum size of 6.843%, and a fermentation time of 120 h. Under these conditions, three replicate validation experiments were performed, and the average lipase activity of *Trichosporon asahii* was 70.816 U/mL. The relative error between the experimental value and the predicted value was only 1.59%, indicating no significant difference between the model prediction and the experimental result. These findings demonstrate that the constructed regression model had high predictive accuracy, stable and reliable results, and strong feasibility and effectiveness for describing the fermentation conditions governing lipase.

## 4. Discussion

Food waste is rich in organic matter, particularly animal and vegetable oils and proteins. During natural composting, it can enrich lipid-utilizing microorganisms, making it an appropriate natural matrix for screening functional lipase-producing strains [2]. In this study, microorganisms were isolated from the oil-water interface of food waste. The results showed that a large tributyrin hydrolysis halo did not necessarily correspond to high lipase activity in olive-oil fermentation, indicating that plate-based D/d values should be verified using liquid assays with long-chain triglyceride substrates.

Yeasts represent one of the most important microbial sources of industrial lipases due to their ability to secrete extracellular enzymes efficiently, relatively mild cultivation requirements, and suitability for large-scale fermentation [26]. Among them, genera such as *Candida*, *Yarrowia*, and *Rhodotorula* have been widely reported for lipase production and industrial applications, particularly in lipid modification and biodiesel synthesis [27,28]. To more accurately demonstrate the advantages of the strain and lipase described in this study, the lipase activities reported for several wild-type microbial strains are summarized below. Wild *Candida* strains cultivated in olive-mill-waste-based media generally showed relatively modest lipase production in several studies, including 9.23 IU/mL for *Candida cylindracea* NRRL Y-17506 and 10.67 U/mL for an olive-mill-waste-derived *Candida tropicalis* isolate, although further optimization in a bench-scale reactor increased the activity of *C. cylindracea* to approximately 20.4–21.6 U/mL [29,30]. Reported extracellular lipase activities of wild-type *Yarrowia lipolytica* vary substantially with strain background, medium composition, inducer type, and activity-assay conditions; representative wild-type cultures have exhibited activities of approximately 49–58 U/cm^3^ by oil-induced cultures [31]. In contrast to the assumption that other *Trichosporon* species possess weak extracellular secretion capacity, wild-type *Trichosporon fermentans* WU-C12 has been reported to produce 126–146 U/mL extracellular lipase, and subsequent studies confirmed the production and purification of multiple extracellular lipase isoforms from this strain [32,33]. Compared with reported wild yeasts, the *Trichosporon asahii* strain reached a higher apparent volumetric activity under shake-flask conditions, while its enzyme also showed alkaline preference and moderate temperature compatibility. These features are favorable for waste-cooking-oil valorization and biodiesel-related biocatalysis.

Microbial lipases typically exhibit broad environmental adaptability, maintaining catalytic activity under diverse conditions such as temperature fluctuations, pH variation, and the presence of organic solvents [34]. The extracellular enzyme produced by *Trichosporon asahii* strains 1-2 showed maximum catalytic activity at 40 °C and pH 8.0, indicating that it is a mesophilic alkaline lipase. This property is advantageous for treating waste cooking oil because mildly alkaline conditions can improve the dispersion of lipid substrates and facilitate enzymatic hydrolysis or subsequent transesterification steps. The enzyme retained more than 80% residual activity after incubation at 30–35 °C for 2 h, suggesting that it is suitable for mild-temperature processing, although its activity decreased at higher temperatures. In addition, K+ and Na+ enhanced activity, whereas Ca^2+^, Mg^2+^, Mn^2+^, Fe^3+^, and EDTA inhibited activity. These results imply that the ionic microenvironment may regulate substrate-interface activation and structural stability of the enzyme, and they also indicate that metal-ion composition should be controlled when crude enzyme preparations are used in waste-oil systems.

Fermentation variables strongly affected extracellular lipase accumulation. Single-factor experiments showed that lipase activity peaked at pH 6.0, 96 h, 6% inoculum size, and 30 °C, whereas response surface analysis indicated that fermentation time had the strongest effect on activity, followed by pH, inoculum size, and temperature. The high activity observed at the late fermentation stage suggests that lipase synthesis is associated with the late exponential or early stationary phase, while excessive inoculum or deviation from the optimal pH may cause nutrient depletion, metabolic stress, or reduced enzyme secretion. Response surface methodology integrated these factors and predicted an optimal combination of 31 °C, initial pH 6.33, 6.843% inoculum size, and 120 h fermentation. Under these conditions, the validated activity reached 70.816 U/mL, representing an approximately 10-fold improvement compared with the initial activity of strains 1-2. This demonstrates that optimization of physical fermentation parameters alone can substantially unlock the production potential of this wild-type *Trichosporon asahii* strain without genetic modification.

The present study evaluated crude extracellular enzyme activity, but did not yet determine purified-enzyme kinetic parameters, substrate specificity toward different waste-oil components, long-term operational stability, or biodiesel conversion efficiency. In addition, although *Trichosporon asahii* can be managed through standardized closed fermentation, biosafety evaluation remains important before scale-up. Future work should therefore focus on enzyme purification, immobilization, pilot-scale fermentation, repeated-batch operation, and direct validation of waste-cooking-oil transesterification performance.

## 5. Conclusions

In this work, a lipase-producing strain of *Trichosporon asahii* was isolated from food waste. The extracellular lipase secreted by this strain displayed maximum catalytic activity at 40 °C and pH 8.0, categorizing it as a mesophilic alkaline lipase. Box-Behnken response surface methodology was applied to tune cultivation parameters for lipase biosynthesis, and the optimized fermentation regime was determined as follows: incubation temperature 31 °C, initial medium pH 6.33, inoculation ratio 6.843%, and a culture period of 120 h. Under this coordinated culture setup, the peak lipase activity reached 70.816 U/mL, marking a tenfold increase compared with the unadjusted basic culture system. The present investigation verifies the inherent lipase-synthesizing capacity of *Trichosporon asahii*, and parameter optimization delivers prominent elevation of enzymatic activity. This finding expands the reservoir of lipolytic microbial strains, and furnishes empirical references for further research on biodiesel production using waste cooking oil catalyzed by this yeast isolate.

## Figures and Tables

**Table 1:** Rescreening results of lipase-producing strains.

Strain	D/d Ratio	Lipase Activity (U/mL)
1-2	2.0	7.165
1-4	2.8	9.290
2-1	1.7	0.550
2-2	1.3	1.778
2-3	2.2	1.373
2-4	1.7	2.583

**Table 2:** Experimental conditions and responses of the four-factor, three-level response surface design for fermentative lipase production.

Run	A: Temperature (°C)	B: pH	C: Time (h)	D: Inoculum Size (%)	Lipase Activity (U/mL)
1	28	5	96	6	9.39
2	32	5	96	6	21.623
3	28	7	96	6	24.797
4	32	7	96	6	56.017
5	30	6	72	4	21.052
6	30	6	120	4	53.882
7	30	6	72	8	38.756
8	30	6	120	8	83.279
9	28	6	96	4	37.626
10	32	6	96	4	41.355
11	28	6	96	8	31.248
12	32	6	96	8	34.098
13	30	5	72	6	14.515
14	30	7	72	6	29.592
15	30	5	120	6	47.211
16	30	7	120	6	66.471
17	28	6	72	6	31.663
18	32	6	72	6	4.86
19	28	6	120	6	30.552
20	32	6	120	6	42.015
21	30	5	96	4	15.477
22	30	7	96	4	22.937
23	30	5	96	8	23.144
24	30	7	96	8	23.37
25	30	6	96	6	38.561
26	30	6	96	6	55.605
27	30	6	96	6	57.071
28	30	6	96	6	57.474
29	30	6	96	6	56.286

**Table 3:** Analysis of variance for the quadratic regression model of fermentative lipase production.

Source of Variation	Sum of Squares	df	Mean Square	F-Value	*p*-Value	Significance
Model	6813.71	14	486.69	2.54	0.0461	*
A: Temperature	100.29	1	100.29	0.5233	0.4813	
B: pH	702.64	1	702.64	3.67	0.0762	
C: Time	2789.90	1	2789.90	14.56	0.0019	**
D: Inoculum size	143.98	1	143.98	0.7513	0.4007	
AB	90.13	1	90.13	0.4703	0.5041	
AC	366.07	1	366.07	1.91	0.1886	
AD	0.1932	1	0.1932	0.0010	0.9751	
BC	4.37	1	4.37	0.0228	0.8821	
BD	13.08	1	13.08	0.0683	0.7977	
CD	34.18	1	34.18	0.1784	0.6792	
A^2^	1341.85	1	1341.85	7.00	0.0192	*
B^2^	1603.02	1	1603.02	8.36	0.0118	*
C^2^	27.46	1	27.46	0.1433	0.7107	
D^2^	296.46	1	296.46	1.55	0.2340	
Residual	2683.10	14	191.65			
Lack of fit	2420.44	10	242.04	3.69	0.1101	
Pure error	262.66	4	65.66			
Corrected total	9496.82	28				

Notes: * represents significant difference at *p* < 0.05; ** represents highly significant difference at *p* < 0.01.

## Data Availability

The original contributions presented in the study are included in the article. Further inquiries can be directed to the corresponding authors.
